# The challenge in diagnosing coarctation of the aorta

**DOI:** 10.5830/CVJA-2017-053

**Published:** 2018

**Authors:** IE Hoffman Julien

**Affiliations:** Department of Pediatrics, University of California, San Francisco, CA, USA

**Keywords:** patent ductus arteriosus, left ventricular failure, pulse oximetry, balloon dilatation, stent

## Abstract

Critical coarctation of the aorta in neonates is a common cause of shock and death. It may be the most difficult of all forms of critical congenital heart disease to diagnose because the obstruction from the coarctation does not appear until several days after birth (and after discharge from the hospital), and because there are no characteristic murmurs. Some of these patients may be detected by neonatal screening by pulse oximetry, but only a minority is so diagnosed. Older patients are usually asymptomatic but, although clinical diagnosis is easy, they are frequently undiagnosed.

Coarctation of the aorta is a congenital lesion that occurs in 2.5 to four per 10 000 live births.^1^,^2^ With a total world population of 7.5 × 109 and an annual crude birth rate of about 1.365 × 106, each year about 340 000 to 550 000 children are born with coarctation of the aorta. The anomaly is usually sporadic and is more frequent in males.

Most coarctations fall into one of two groups: critical coarctation of the aorta that causes symptoms within two months of birth and if untreated causes death, and asymptomatic coarctation of the aorta that presents later, usually with hypertension in the upper limbs. Critical coarctation of the aorta accounts for about 60% of all coarctations.

## Pathological anatomy

**Fig. 1:**
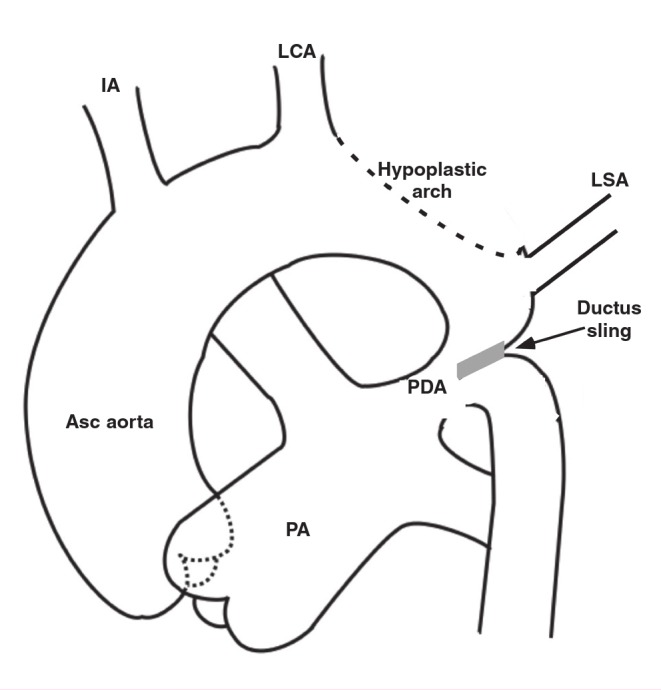
Basic anatomy of coarctation of the aorta. Asc: ascending; IA: innominate artery; LCA: left carotid artery; LSA: left subclavian artery; PA: main pulmonary artery; PDA: patent ductus arteriosus.

Anatomically a coarctation of the aorta is a shelf of tissue extending from the postero-lateral aortic wall towards the ductus arteriosus (Fig. 1). The shelf is near the patent ductus arteriosus, sometimes above or below it, and is better termed juxtaductal. A sling of ductus muscle passes around the shelf, and more ductus muscle extends into the aortic wall above and below the shelf. This is important, because unlike most other smooth muscle, ductus smooth muscle tends to contract when exposed to high oxygen concentrations.

The arch is often hypoplastic and when present, an associated intracardiac shunt should be suspected. Most commonly this is an isolated ventricular septal defect, but almost any form of complex congenital heart disease can be associated. About 50 to 70% of the patients have a bicuspid aortic valve. The left subclavian artery is often hypoplastic, and in approximately 5% of these patients arises distal to the shelf.

## Developmental physiology

In the foetus, the patency of the ductus arteriosus depends on a balance between constrictors and dilators. Constriction is due mainly to an increased sensitivity of ductus smooth muscle to calcium^3^ but also to endothelin. By contrast, the ductus smooth muscle is relaxed by vasodilator prostaglandins (mainly PGE_2_) that are produced in the ductus wall and also circulate from the placenta.^4^ The PGE_2_ increases intracellular concentrations of cAMP, which decreases calcium sensitivity. Nitric oxide and carbon monoxide may play minor roles. In addition to these chemical factors, the high pressure in the ductus lumen helps to keep it open.

After birth, the lowered pulmonary vascular resistance lowers pressure in the ductus lumen, and PGE_2_ decreases both from loss of placental prostaglandins and a reduced number of PGE_2_ receptors in the ductus wall. The increase in arterial oxygen content has several actions that favour constriction of the ductus. A membrane-bound cytochrome 450 acts as a transducer to produce vasoconstrictors.^5^ Oxygen inhibits potassium channels, produces membrane depolarisation, increases smooth muscle calcium, and induces the formation of endothelin-1; all these changes stimulate vasoconstriction, although the role of endothelin-1 is not clear.

## Pathophysiology

**Fig. 2:**
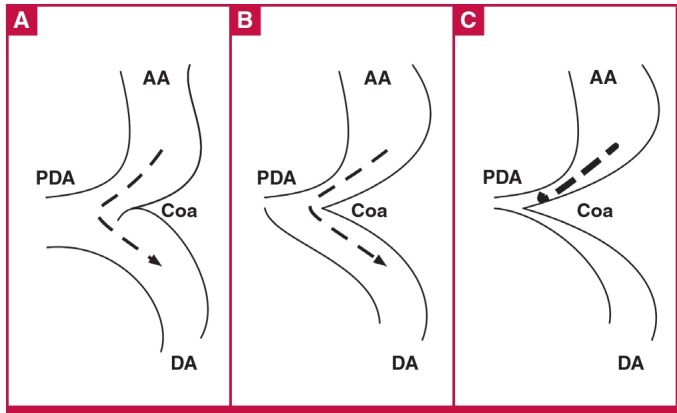
A: At birth, the ductus (PDA) is wide open, so that despite the coarctation shelf (Coa), flow is not obstructed. Dashed line with arrow shows unimpeded flow. B: Ductus closes at its connection to the main pulmonary artery, but its ampulla still provides a detour for flow. Dashed line with arrow shows unimpeded flow. C: Narrowing of the ductus ampulla leads to severe flow obstruction. Heavy dashed line shows obstructed flow. AA: ascending aorta; DA: descending aorta.

At birth, the ductus is wide open so that despite the shelf, blood can flow freely from ascending to descending aorta (Fig. 2A). The ductus closes first at its pulmonary arterial end, but the wide ductus ampulla provides space for unobstructed flow (Fig. 2B). This region then narrows further as the ductus ampulla shrinks and the ductus sling contracts, drawing the lateral wall towards the closing ampulla (Fig. 2C).^6^,^7^

As a rule of thumb, an artery must be narrowed by more than 50% before any obstruction occurs, but once this degree of narrowing occurs, it takes very little additional narrowing to produce severe obstruction, which can occur very rapidly. (This functional narrowing can be reversed by infusing PGE_1_.^7^,^8^) The sudden severe obstruction overloads the left ventricle, causing left ventricular failure, pulmonary hypertension, right ventricular failure and systemic congestion. Pulmonary oedema is often seen.

If the foramen ovale is patent there will be a left-to-right atrial shunt that can be large, and if it occurs there may be no or less pulmonary oedema, because left atrial pressure is lower. The patient often goes into shock. If the narrowing of the aorta occurs very slowly, the left ventricle has time to adapt to the increased pressure load and develop a collateral circulation and shock and congestive heart failure do not occur. These patients are usually asymptomatic and are diagnosed at older ages.

## Diagnosis: critical coarctation of the aorta in neonates

Coarctation of the aorta is a treacherous disease that is often undiagnosed. When neonates present with shock, the most common cause is sepsis, followed closely by left heart obstruction (aortic stenosis, coarctation of the aorta), which must always be excluded. These cardiac lesions usually have insignificant and non-diagnostic murmurs. The electrocardiograms show right ventricular hypertrophy, not left ventricular hypertrophy as expected from a left heart obstructive lesion. A chest radiogram will show a dilated heart and either pulmonary oedema or a leftto- right shunt. The liver is usually enlarged.

Recent studies done in Scandinavia found that at least 50% of these neonates were discharged without a diagnosis,^9^ and that most were still undiagnosed at five days after birth.^10^ In California, Chang et al.^11^ found that 27% of patients with coarctation of the aorta died undiagnosed at a median age of 17 days. Ward et al.^12^ observed that infants with symptomatic coarctation of the aorta presented between five and 14 days after birth.

Coarctation can be detected by foetal echocardiography if performed by an expert, but even then is often not detected.^9^ This is therefore not a diagnostic method for general use.

In older patients the main physical signs are hypertension in the arms and weak, delayed femoral arterial pulses. These signs however, are either not detected or are unreliable in neonates because in most of the infants there is no obstruction to flow immediately after birth, and hypertension or weak femoral pulses may take several days to appear. Feeling femoral pulses may be difficult, especially in plump babies, and by the time decreased femoral pulses are obvious, the obstruction is fairly severe. Taking four-limb blood pressures in normal neonates is not routine, and even if performed, the pressures may not be accurate. If blood pressures are taken, use the right rather than the left arm.

When pulse oximetry screening for critical congenital heart disease in neonates was introduced, several patients with coarctation of the aorta were detected because they had a rightto- left shunt through the patent ductus arteriosus. Unfortunately, this occurs in only a minority of coarctations, perhaps related to the anatomy of the region, so that pulse oximetry cannot be relied upon for the diagnosis.^9^,^13^

At present, there is no easy way to make this diagnosis in a timely fashion. The best way is to have all neonates seen between three and seven days after birth by a physician or nurse who can check the femoral pulses. If these are found to be decreased then immediate transfer to a hospital for treatment should be done. Waiting is not an option because the likely course is rapid deterioration. If the patient is already symptomatic, an infusion of prostaglandin PGE_1_ at 0.05 to 0.15 mcg/kg/min will help to relax the ductus muscle and reduce the obstruction, relieve the symptoms, allow the left ventricle to recover, and make definitive treatment safer.

## Diagnosis: coarctation of the aorta in older patients 

Although the diagnosis of coarctation of the aorta in older patients is easy, the diagnosis is often missed or delayed. In fact, even in older children, coarctation of the aorta is missed in about 85% of coarctation patients referred to a hospital for murmurs or hypertension.^12^,^14^-^16^

In these patients, the left ventricle has adapted to the pressure load with hypertrophy, and lower body perfusion is achieved by hypertension in the upper aorta. Most are asymptomatic until later in life, although a few may have pain on walking (intermittent claudication). If left untreated, 50% die under 30 years of age and few survive beyond 50 years of age. Death is due to congestive heart failure, dissection and rupture of the aorta, ruptured berry aneurysm in the cerebral circulation, or infective endocarditis.

The clinical diagnosis is based on finding signs of left ventricular hypertrophy, hypertension in the upper body, weak and delayed femoral arterial pulses, and upper body collateral circulation.

- Left ventricular hypertrophy is appreciated by a forceful, heaving apical thrust in systole.
- Blood pressure should be taken with an appropriate-sized cuff in both arms because the left subclavian artery is often hypoplastic or distal to the coarctation, and in more than 1% of people, the right subclavian artery is aberrant and could originate below the coarctation. In these people the pressure will be higher in the left than the right arm.
- If the arm pressure is high, then feel the femoral pulses. In coarctation of the aorta they are weaker than the radial pulses, and when felt simultaneously with the radial pulse, the femoral pulse is delayed. Confirmation is obtained by obtaining a leg blood pressure with an appropriate-sized cuff.
- Collateral arteries are perceptible in over 50% of these patients, and are prominent over the edges of the scapulae.
- An ejection-type systolic murmur may be heard at the base, and between the spine and the left scapula.
- Confirmation of the diagnosis can be obtained by a chest radiogram that will show the aortic indentation at the coarctation site (three sign) and rib notching of the lower ribs due to enlarged intercostal arteries. Echocardiography is indicated if additional cardiac lesions are suspected.

## Treatment

There are two main treatment choices: surgery or balloon dilatation with stenting.^17^,^18^ A variety of surgical procedures has been used, but most common are mobilising the aorta, excising the region around the coarctation and reconnecting the two ends, or mobilising the aorta, excising the region around the coarctation and connecting the descending aorta to the underside of the aortic arch. The latter is the preferred technique in infants with a hypoplastic arch because it allows reconstruction of the aortic arch.

The operative mortality rate is very low. There is a small incidence of paraplegia with surgery unless precautions are taken to prevent spinal cord ischaemia. With current surgical methods there is a 3–5% chance of re-coarctation. Surgery in older adults has a higher mortality rate because of an abnormal aortic wall and the development of huge, thin-walled intercostal aneurysms.

An alternative is to dilate the coarctation with a balloon and then insert a stent to prevent the dilated region from narrowing due to elastic recoil. Usually infants are treated surgically because they are too small for introducing the stent on a catheter, whereas balloon dilatation and stenting are usually used in adults and for treating re-coarctation. All procedures carry a small risk of aneurysm formation.

## Prognosis

In general, the results are good, although coarctation patients had a greater and earlier incidence of cardiovascular disease than the normal population.^19^,^20^ In addition, although blood pressure decreases after removal of the obstruction, a high proportion of these patients develop persistent hypertension.^19^,^21^-^23^

## Conclusions

The timely diagnosis of critical coarctation of the aorta in neonates is difficult, and data from Scandinavia, Europe and the USA show how poorly this is done. Delayed diagnosis also occurs in older patients, despite the ease of diagnosis.

These problems are likely to be worse in Africa with a widely dispersed largely rural population and a shortage of medical resources. Furthermore, with the high average birth rate in Africa, the present population of 1.2 billion is expected to double by 2050, thereby increasing the number of children born with congenital heart disease in general and coarctation of the aorta in particular.

Nevertheless, attempts should be made to diagnose and treat coarctation of the aorta at any age, especially because diagnosis needs only good physical examination. Treatment of coarctation of the aorta is cheaper, easier, and results are better than for most other forms of critical congenital heart disease, and most patients do well after the obstruction is removed.
